# 

**Published:** 1889-02-09

**Authors:** 


### Notice to Advertisers and Subscribers.

All Advertisements, Orders for Copies of The Hospital, and Business Communications should be addressed to The Publisher, *not to the Editor*, The Hospital, 38, Old Jewry, E.C.

### To Residents, Secretaries, and Matrons.

The Editor will be glad to have the Regulations and each Report as issued by Asylums, Hospitals, Convalescent and Nursing Institutions, as well as those of other Charities, and an early copy in duplicate of all Appeals and Festival Notices, etc., addressed to *The Editor, The Lodge, Porchester Square, London, W.* Any superintendent, secretary, matron, or other chief official who regularly sends such information may be registered, on application to the Publisher, to receive free of cost a cover for binding The Hospital in half-yearly volumes.

A Scale of Charges will be found on the first page of the Nursing Supplement.

### Scraps and Gleanings.

Mr. John Bright still sticks to homœopathy.

Glasgow Lock Hospital treated 186 patients last year.

It is proposed to build a Gordon Mission Hall at Plumstead.

Mrs. Perrin's Convalescent Home at Malvern Link is now open.

The Hahnemann Hospital, Liverpool, reports a deficiency of £500.

Dr. Flynn has been appointed Medical Officer of Cork Dispensary.

The paupers in London just now number 200 less than at this time last year.

The London cabmen have contributed £123 to the Hospital Saturday Fund.

Dr. C. M. Tidy has been awarded the Survey prize of a hundred guineas.

The Chelsea Hospital for Women is going to have a convalescent home of its own.

The Extension Fund of Bradford Children's Hospital now exceeds £6,000.

There were 83 deaths from measles in London last week, and 33 from diphtheria.

Mr. Isaac Wilson, M.P., opened a bazaar last week in aid of the North Riding Infirmary.

The recent concert at Port Glasgow resulted in a sum of £30 for the Greenock Eye Infirmary.

The Duke of Westminster presided at the annual meeting of the Chester Infirmary last week.

On January 25th a ball was held at Preston in aid of the Preston and Lancaster County Infirmary.

The Hunterian lectures will be delivered on February 22nd, March 1st, 8th, and 15th, by Mr. T. Holmes.

Derbyshire General Infirmary is to have a new disinfector from Messrs. Manlove, at a cost of £216.

The Brighton Throat and Ear Hospital issues a satisfactory report. They have a balance of £43 in hand.

The Belgian Academy of Medicine has issued a professional interdiction against the practice of hypnotism.

Typhus is prevalent amongst our troops in Egypt. Four men of the 17th Soudanese have died of this disease.

A Cinderella dance was given at the Portman Rooms on January 31st, in aid of the New Hospital for Women.

Alderman Banks gave a dinner to the members of the Coventry Workmen's Hospital Saturday Fund last week.

The Queen has personally inspected the new hospital ship *Victoria*, which was towed to Portsmouth especially for her to see.

Mr. Alderman Moscrop, late Mayor of Bolton, has bequeathed £2,000 to Bolton Infirmary, and £13,000 to other charities.

Birmingham Children's Hospital treated 14,000 patients last year. Of 13 cases of diphtheria requiring tracheotomy, 6 recovered.

The new rules for Hereford Infirmary number 76. The rules of St. George's Hospital number 290, and those of Northampton 213.

The Torbay Hospital and Provident Dispensary is not in debt. Its supporters should do their best to maintain this unusual state of affairs.

It is proposed to pay a year's salary to the widow of the late Dr. Golding, of Cork, who lost his life through the unsanitary state of his district.

The Queen has made a grant of cast linen to the Mission to Deep Sea Fishermen, for use on board the pioneer cruising hospital *Queen Victoria*.

The report of the Salisbury Provident Dispensary reads—Rent, 5s. This is owing to the generosity of Dr. Roberts, who practically gives the premises.

The Duke of Cambridge will preside at a festival dinner on behalf of the Building Fund of the City Orthopædic Hospital, on Wednesday, the 26th of June.

The King of the Belgians has founded a much-needed African Red Cross Association. Sanitary matters need much attention in that unhealthy country.

Sligo Infirmary issues a good report. Dr. M'Dowell, the house surgeon, attended 338 externe patients last year, besides the usual number of in and out-patients.

"An Old Etonian" writes to the *Post* that he thinks, while improvements are being made at Eton, they should include a sanatorium in charge of trained nurses.

At the fourteenth annual meeting of Grantham Hospital it was stated that a considerable sum has already been collected for the new children's wards which have just been opened.

At the annual meeting of Rotherham Hospital subscribers last week, the Rev. Dr. Falding was presented with a silver salver bearing £100, as a recognition of aid given to that and other charities.

According to the Exchange Telegraph Company, the College of Physicians at their last meeting passed a vote of censure on Sir Morell Mackenzie for publishing his book on Frederick the Noble.

The Tuesday and Thursday lectures at the Royal College of Physicians will include the Milroy lectures by Dr. Arlidge, the Goulstonian lectures by Dr. Tooth, and the Lumleian lectures by Dr. John Harley.

At the annual meeting of the Liverpool Hospital for Women, Mrs. Alfred Booth made a long speech in favour of Dr. Imlach. We fancy we have not yet heard the last of Dr. Imlach and his grievances against this hospital.

Miss Ryland, of Barford, who died on January 28th, has left £36,000 to charities and £300,000 in personal legacies. Her benefactions include gifts of £1,000 each to the Queen's and the Women's Hospitals, Birmingham, Warneford Hospital, the Home for Incurables, Leamington, and the General Hospital, Birmingham, with a further £25,000 to the latter institution on condition that a new hospital is erected within five years.

### Amusements and Relaxation.

### NEW PUZZLE PRIZE SCHEME.

SECOND QUARTERLY COMPETITION COMMENCED JANUARY 5TH, ENDS MARCH 30TH, 1889.

1. Prizes will be awarded for the best Original Puzzles in Prose or Verse relating to any of the Social, Political, or Philanthropical topics of the day. Competitors are requested to *strictly* observe this rule, or their contributions will be discredited.
2. Puzzles may take any form which the ingenuity of the competitors suggest. Answers must accompany contributions, and the source of obscure or ambiguous terms be given.
3. No competitor will be allowed to take more than one prize during the Quarter.
4. Eight Prizes of Standard Books will be given, the choice of the book to be left to the winner. First, Second, Third, Fourth, and Fifth Prizes for best Original Puzzles, £1 1s., 15s., 10s. 6d., 7s. 6d., and 5s. each.
5. First, Second, and Third Prize for Solutions of 15s., 10s. 6d., and 5s. each.

N.B.—All contributions must reach the office, 38, Old Jewry, E.C., not later than the *First Post* on *Thursday* in each week.

### PUZZLES FOR SOLUTION.

**Sixth Week.**

No. 12. Double Acrostic.

Ere long, as my primals it hath willed
My finals, will with English folk be filled.

1. The only light that here must fly
Beyond the limits of geography.
2. "Great River" with a Delta spreading wide—
Forests and jungles lining every side.
3. Island, which bears the memory of a name
Whose wild ambition nought but death could tame.
4. Mountain o'erlooking the quiet strand
Of a peaceful lake in the Holy Land.
5. River, utilised throughout its course,
Though marshy, midway from its source.
6. An executive district bears this name;
An island also is called the same.
7. A military stronghold this,
And on Bavarian soil I wis.
8. Town within the torrid zone,
Peopled by negro tribes alone.

Tinie.

No. 13. Diamond Puzzle.

1. A consonant, whose pronunciation
We hear that which divides our British nation.
2. For this filled oft by little lad or maid,
Let our subscriptions willingly be paid.
3. The sympathy we feel for these we know in need,
How oft our editor must wish was not in word but deed.
4. Give these in answer to the needy's call,
Many they help, although of value small.
5. Comfort to weary mothers, who earn their daily bread,
Two meanings has this word—here, silent lives are led.
6. Our intellect and interest here combined
Form pleasant occupation for the mind.
7. Working mid all men, righting wrong;
Pain without thee, we suffer long.
8. To those who suffer aching pain a momentary cure;
Some doctors disapprove and say, "'Tis better to endure."
9. No surgeon is without this light—*it* nurses also need,
And artisans if they would wish success in every deed.
10. From this a disagreeable but wholesome fluid comes,
And doctors often order its use in many homes.
11. This consonant you soon will guess, if other lights are clear,
And when you've added it to them, the answer will appear.

Cotonopolis.

**Marks for Original Puzzles.**

| Names. | No. of Puzzles sent in Jan. 31. | No. of Marks Jan. 31. | Totals. | Names. | No. of Puzzles sent in Jan. 31. | No. of Marks Jan. 31. | Totals. |
|---|---|---|---|---|---|---|---|
| Cotonopolis... | 1 | 3 | 19 | Padre ......... | — | — | 3 |
| Tinie............ | 3 | 5 | 21 | Prior........... | 3 | 2 | 9 |
| Jenny Wren . | — | — | 4 | Lightowlers . | 2 | 5 | 9 |
| Patience ...... | 3 | 6 | 21 | Twentieth ... | — | — | 3 |

**Answers.**

No. 8. Double Acrostic.

D iade M
O per A
C ara T
T owe R
O thell O
R ecitatio N

No. 9. Triple Acrostic.

C ro W di E
O ph I di A
L io N es S
D ea D se T

**Marks for Solution of Puzzles.**

Total Marks.—Flora (1), 3; Mater, (1), 1; Scrooge (1), 3; Patience, 0; Lady Clara (0), 3; Ruffles (0), 2; Lauriston, 0; Reldas, 0; Jenny Wren, 2.

[*The Figures in parentheses are Marks for the week ending Jan. 31st.*]

**Conditions.**

I. Every paper sent in must be clearly written, and one side only must be used. All competitors must mark at the head of their papers the number of their competition.

II. Each paper must be signed by the author with his or her real name and address. A *nom de plume* may be added if the writer does not desire to be referred to by us by his real name. In the case of all prizewinners, however, the real name and address will be published.

III. All communications relating to prize competitions should be addressed to "The Prize Editor, The Hospital, 38, Old Jewry, London, E.C." Competitors are requested not to include in letters, etc., to the Prize Editor, communications on matters of other business. All letters must be fully prepaid.

IV. The Prize Editor of The Hospital is the sole judge of the competitions, and his decision is in all cases final and without appeal.

V. The Prize Editor reserves the right to publish any paper sent in, whether it receives a prize or not. He does not hold himself responsible for any MSS. sent, nor can he undertake to return rejected competitions.

